# Patterns and Sociodemographic Characteristics of Substance Abuse Among the Adult Population in Makkah City, Saudi Arabia: A Cross-Sectional Study

**DOI:** 10.7759/cureus.46573

**Published:** 2023-10-06

**Authors:** Omar F Alharbi, Abdullah S Alharbi, Abdullah A Alsubhi, Fawaz S Baalaraj, Abdullah E Alharbi, Salem B Basulayman, Bayan Z Fatani, Omar Babateen, Abdullah Tawakul

**Affiliations:** 1 Department of Medicine and Surgery, College of Medicine, Umm Al-Qura University, Makkah, SAU; 2 Mental Health Department, Neuroscience Center, King Abdullah Medical City, Makkah, SAU; 3 Department of Physiology, College of Medicine, Umm Al-Qura University, Makkah, SAU; 4 Department of Medicine, College of Medicine, Umm Al-Qura University, Makkah, SAU

**Keywords:** depression, stress, anxiety, cannabinoids, substance abuse, smoking, psychoactive substance, saudi arabia, makkah

## Abstract

Background: Substance abuse is a term that refers to the harmful or hazardous use of psychoactive substances, including alcohol and illicit drugs. One of the key impacts of illicit drug use on society is the negative health consequences experienced by its members.

Objective: This study recorded the pattern of substance abuse and the sociodemographic characteristics of adult substance abusers in Makkah City.

Methods: An online self-administered survey was provided to the general population through social media platforms between March 2023 and August 2023. Males and females living in Makkah over the age of 18 were included in it. The participants who refused to take part or those who were younger than 18 were not included in the study.

Results: The number of participants in this study was 720; 73.5% were under the age of 30 and 424 were females (58.9%). The significant variables between substance abuse and sociodemographic data were gender (P=0.001), depression (P≤0.000), anxiety (P≤0.000), stress (P=0.025), and bad/shocking experience during childhood (P=0.004).

Conclusion: Substance abuse positively correlates with sociodemographic data, with males having a higher risk, and psychiatric neurosis is associated with childhood trauma and anxiety.

## Introduction

Substance abuse is a term that refers to the harmful or hazardous use of psychoactive substances, including alcohol and illicit drugs; one of the key impacts of illicit drug use on society is the negative health consequences [1]. Substance abuse additionally places a significant financial burden on people, families, and society [1]. Substance abuse is a major pandemic around the world; according to the United Nations Office on Drugs and Crime, there were over 36 million individuals who had drug use disorders in 2021, leading to massive effects on population-wide health and socioeconomic status [2]. Thus, health workers should be able to identify substance abuse patterns and sociodemographic characteristics to face this pandemic [2].

Despite the limited information regarding substance abuse in Saudi Arabia, there are some data that illustrate its effect on the Saudi population. Indeed, several studies have been conducted on a Saudi population and reported some information about specific substances. One study assessed the prevalence of smoking in Saudi Arabia, with 10,735 participants in total, representing 13 different regions of the entire country; half were men and half were women [3]. This study showed a 12.2% overall prevalence of smoking [3]. Another retrospective study done in Al-Amal Hospital in Jeddah targeted patients who were followed up in clinics; of the 17,254 male and 10,562 female patients, 78 (16.1%) were alcohol abusers [4]. Another review mentioned that the most common illicit drug abused by Saudis in addiction treatment settings was amphetamine (4-70.7%) [5]. There was an increase in using amphetamine during the past 10 years, along with opioid abuse [5]. A review article in Jeddah regarding substance abuse in Saudi Arabia showed that among Saudi patients in addiction treatment settings, 6.6-83.6% were heroin abusers [6]. Amphetamines, heroin, alcohol, and cannabis were the most commonly abused substances [6]. The review included many studies concluding that cannabis use ranges between 1% and 60% [6].

Our study is one of the few studies on substance abuse in Saudi Arabia. This work recorded the pattern of substance abuse and the sociodemographic characteristics of adult substance abusers in Makkah City. We also attempted to cover the multi-substance abuse prevalence in the same population target.

## Materials and methods

Study design and participants

The purpose of this cross-sectional observational study was to determine the pattern of substance abuse and the sociodemographic characteristics of adult substance abusers and also assess multi-substance abuse prevalence in Makkah, Saudi Arabia. Convenience sampling was used for participant recruitment. Males and females living in Makkah over the age of 18 were included. The participants who refused to take part or those who were younger than 18 were excluded.

Ethical considerations and sample size

An online self-administered survey was provided to the general population through social media platforms between March 2023 to August 2023. Ethical approval was obtained from Umm Al-Qura University’s Biomedical Ethics Committee (approval No. HAPO-02-K-012-2023-02-1477). Here, 385 was the minimum number of participants needed for the sample size with a 95% confidence interval and a p-value of 5% as calculated by the OpenEpi software (version 3.01) [7]. A total of 720 participants were recruited for this study to enhance the generalizability and precision of the findings.

Study tool

The questionnaire was composed of three parts: The first part gathered sociodemographic data, and the second part consisted of 11 multiple-choice questions, including the Addiction Severity Index (5th edition) [8]. The last part included the Depression Anxiety Stress Scale (DASS) [9]. The DASS score ranged from normal to mild, moderate, severe, and extremely severe. These ranges respectively represent the values of 0-4, 5-6, 7-10, 11-13, and ≥14 for depression; 0-3, 4-5, 6-7,8-9, and ≥10 for anxiety; and 0-7,8-9, 10-12, 13-16, ≥17 for stress [10]. In addition, a pilot study was conducted on 40 participants to guarantee clarity and simplicity of the questionnaire. The 40 participants were excluded from the results. The survey validity was assessed by two independent public health and community medicine specialists in the previous study [11].

Statistical analysis

The analysis used IBM SPSS Statistics for Windows, version 26 (released 2019; IBM Corp., Armonk, New York, United States), and categorical data were presented as frequencies and percentages. A chi-squared test assessed the association between two categorical variables, and multiple logistic regression analysis was done to assess the impact of several factors on substance abuse.

## Results

As shown in Table 1, there were 720 participants in this study; 73.5% were under the age of 30, and 424 were female (58.9%). Most participants (79.4%) had university or higher education, and 56% of them were students. The vast majority of the participants were singles (70.6%). Our population indicated that 65.1% did not have any bad/shocking experience during their childhood. Most (83.2%) did not use any drugs during the last year (including tobacco products). Of the 720 participants, 20.6% of them had depression, 21.3% had anxiety, and 7.5 % had stress.

**Table 1:** Characteristics of the participants

Variable	Choices	Frequency	Percentage
Age	<30	529	73.5%
	30 or higher	191	26.5%
Gender	Female	424	58.9%
	Male	296	41.1%
Education	Uneducated	5	0.7%
	High school or below	143	19.9%
	University or higher education	572	79.4%
Employment status	Student	403	56.0%
	Unemployed	105	14.6%
	Employed	212	29.4%
Marital status	Single	508	70.6%
	Married	177	24.6%
	Separated/widow	35	4.9%
Did you have any bad/shocking experience during your childhood?	No	469	65.1%
	Yes	251	34.9%
Did you use any drugs during the last year (including tobacco products)	No	599	83.2%
	Yes	121	16.8%
Depression	No	572	79.4%
	Yes	148	20.6%
Anxiety	No	567	78.8%
	Yes	153	21.3%
Stress	No	666	92.5%
	Yes	54	7.5%

Table 2 shows that 85.7% of the participants did not use cigarettes (including electronic cigarettes), and only 3.8% used chewing tobacco. The vast majority of them (89.9%) were not using shisha (hookah); only 16 (2.2%) were using khat (*Catha edulis*). The same results can be seen in Table 2 for the number of the participants who did not use sedatives/hypnotics, cannabis, and alcohol (98.3%, 97.6%, and 98.6%, respectively). Most participants (99.6%) stated that they did not use amphetamines, and only two people were using methamphetamines (0.3%). The majority were not using cocaine, opioids, and ecstasy (MDMA) (99.9%, 99.4%, and 99.7%, respectively).

**Table 2:** Numbers and percentages of drug abusers

Variable	Choices	Frequency	Percentage
Cigarettes (Including electronic cigarettes)	No	617	85.7%
	Yes	103	14.3%
Chewing tobacco	No	693	96.3%
	Yes	27	3.8%
Shisha (hookah)	No	647	89.9%
	Yes	73	10.1%
Khat (*Catha edulis*)	No	704	97.8%
	Yes	16	2.2%
Sedatives/hypnotics	No	708	98.3%
	Yes	12	1.7%
Cannabis	No	703	97.6%
	Yes	17	2.4%
Alcohol	No	710	98.6%
	Yes	10	1.4%
Amphetamines	No	717	99.6%
	Yes	3	0.4%
Methamphetamine	No	718	99.7%
	Yes	2	0.3%
Cocaine	No	719	99.9%
	Yes	1	0.1%
Opioids	No	716	99.4%
	Yes	4	0.6%
Ecstasy (3,4-methylenedioxy-N-methamphetamine or MDMA)	No	718	99.7%
	Yes	2	0.3%

Table 3 shows that there were 165 participants aged 30 or above who did not use any substances (86.4%). There were 82 males who used substances (27.7%) and 39 females (9.2%). There were 477 (83.4%) participants who had university or higher level of education and who did not have substance abuse. Among the employed participants, 41 of them (19.3%) used substances; 20% of the separated/widowed participants had substance abuse, while 80% did not experience any substance abuse. Of the participants who had bad/shocking experiences during their childhood, 56 people (22.3%) had substance abuse. Among the participants who had depression, 40 participant (27%) had substance abuse; 29.4% of the participants who had anxiety had used substances. Of those who experienced stress, 27.8% of them had substance abuse. The significant variables between substance abuse and sociodemographic data were gender (P=0.001), depression (P≤0.000), anxiety (P≤0.000), stress (P=0.025), and bad/shocking experience during their childhood (P=0.004).

**Table 3:** Association between substance abuse and each of the demographic factors and psychological factors

		Substance abuse				
		No		Yes		
		Frequency	Percentage	Frequency	percentage	P-value
Age	<30	434	82.0%	95	18.0%	0.169
	30 or higher	165	86.4%	26	13.6%	
Gender	Female	385	90.8%	39	9.2%	<0.001
	Male	214	72.3%	82	27.7%	
Education	Uneducated	5	100%	0	0.0%	0.543
	High school or below	117	81.8%	26	18.2%	
	University or higher education	477	83.4%	95	16.6%	
Employment status	Student	343	85.1%	60	14.9%	0.300
	Unemployed	85	81.0%	20	19.0%	
	Employed	171	80.7%	41	19.3%	
Marital status	Single	418	82.3%	90	17.7%	0.388
	Married	153	86.4%	24	13.6%	
	Separated/widow	28	80.0%	7	20.0%	
Did you have any bad/shocking experiences during your childhood?	No	404	86.1%	65	13.9%	0.004
	Yes	195	77.7%	56	22.3%	
Depression	No	491	85.8%	81	14.2%	<0.000
	Yes	108	73.0%	40	27.0%	
Anxiety	No	491	86.6%	76	13.4%	<0.000
	Yes	108	70.6%	45	29.4%	
Stress	No	560	84.1%	106	15.9%	0.025
	Yes	39	72.2%	15	27.8%	

There was a significant positive association between substance abuse and male gender (odds ratio (OR)=5.010, P≤0.001), bad/shocking experiences during childhood (OR=1.661, P=0.026), and anxiety (OR=2.884, P≤0.001), as shown in Table 4.

**Table 4:** Multiple logistic regression analysis of possible factors associated with substance abuse CI: confidence interval

			95% CI		
Variable	Choices	Odds ratio	Lower	Upper	P-value
Age	30 or higher	0.692	0.418	1.146	0.153
Gender	Male	5.010	3.188	7.872	<0.001
Bad/shocking experiences during childhood	Yes	1.661	1.063	2.596	0.026
Depression	Yes	1.172	0.638	2.152	0.609
Anxiety	Yes	2.884	1.599	5.200	<0.001
Stress	Yes	0.942	0.433	2.047	0.880

## Discussion

This study describes substance abuse features among the adult population in Makkah City, i.e., sociodemographic data and patterns of the abuse. The motivation was a significant knowledge gap on this topic [5].

In our study, most substance abusers were under the age of 30. Surprisingly, 58.9% of them were female, and most had university or higher education level. The overall substance use (including tobacco products) is estimated at 16.8% of the 720 participants. Around 28.2% of the 720 participants used tobacco products, which is more than double the prevalence of smoking in Saudi Arabia, accounting for 12.2% [3]. The three types of tobacco products included could explain the higher prevalence among our population. The prevalence of abusing illicit drugs other than tobacco products in Makkah seems to be higher by 9.4% among the 720 participants.

Meanwhile, 5.8% of the worldwide global population is aged 15-64 [12]. Cigarettes (including electronic cigarettes) were the most frequent comorbid substance, followed by shisha (hookah) and chewing tobacco. In comparison, in a cross-sectional study that was conducted on several universities in Egypt, 117 among the 2,380 participants were drug addicts, 4.9% of the drug addicts reported cannabinoid abuse and 41% reported smoking cigarettes, and the most used substance was hashish, followed by strox [11]. Young adults, aged 18 to late 20s, are often the most common substance users, with high rates of alcohol and substance misuse. A survey by the Substance Abuse and Mental Health Services Administration in the United States found that 35% of people aged 18 to 25 binge-drank in the year of 2018, with 25% of people aged 26 and older binge-drinking. Young adults, particularly between the ages of 21 and 22, are also more likely to use illicit substances, such as marijuana, amphetamines, cocaine, hallucinogens, and MDMA [13].

Non-college youths have higher rates of use of almost all illicit substances compared to college students [14]. Men and women with sexual minority status are at a higher risk of developing alcohol, drug, or tobacco abuse and dependence [14]. The use of certain drugs among young people is increasing, with marijuana use among 19- to 28-year-olds reaching all-time highs in 2019 [14]. In Kenya, a total of 9,742 high school, college, and university students were chosen randomly in four cities that were engaged with the Africa Mental Health Research and Training Foundation to be participants in the study [15]. Majority of the participants were males, and alcohol abuse was the commonest and inhalants were the least among the study sample [15].

In Aseer Region, a retrospective study included 8,750 subjects with amphetamine abuse at 16.01% and cannabis at 9.92%, in which younger and single individuals are more likely to use amphetamine, with age and marital status significantly influencing the type of drug [16]. Another study conducted in the Jazan region found that among a total sample of 10,000 participants, 3.8% are female khat chewers and 37.70% are male khat chewers, with an overall 21.4% prevalence of khat chewing in the population (colleges 15.2% vs. schools 21.5%) and significant differences based on age, gender, and residence across different colleges and provinces [17]. Cannabis takes second place in our study after nicotine by 2.4%, followed by khat (around 2.2%). Meanwhile, the prevalence of other substances, such as sedatives/hypnotics, alcohol, opioids, amphetamines, methamphetamines, ecstasy, and cocaine, is 1.7%, 1.4%, 0.6%, 0.4%, 0.3%, 0.3%, and 0.1%, respectively.

Psychiatric comorbidities, including depression, anxiety, and other stressors, had a significant association with substance abuse with a total of 356 participants with positive psychiatric comorbidity. There is a significant positive association between substance abuse and male gender (OR=5.010, P≤0.001), bad/shocking experiences during childhood (OR=1.661, P=0.026), and anxiety (OR=2.884, P≤0.001). As showed in a randomized controlled trial of 103 individuals within Greater Sydney, Australia, 77% of individuals treated for substance use disorder and post-traumatic stress disorder experienced at least one childhood trauma [18]. With lifetime rates of 28.8% for anxiety disorders and 14.6% for drug use disorders, these mental conditions are common in the United States, and a high prevalence of co-occurring disorders has been found, according to the National Epidemiologic Survey on Alcohol and Related Conditions [19]. The National Institute of Health Sciences estimates that one-third of patients with serious depression also struggle with alcoholism; drug or alcohol addiction makes depression more common since these addictions can make people feel more isolated, depressed, and hopeless. Clinical depression affects a person's capacity to work and uphold a healthy lifestyle for weeks, months, or even years at a time. Over time, substance addiction can worsen the effects of depression and cause health issues, such as brain damage [20].

There are various limitations to our study. First, Makkah City lacks a specialized addiction center, which can affect the general population of the city. Second, our study was conducted electronically, which may have impaired the participants’ understanding and our inability to understand the motivations behind responders and reasons of other people's refusals. Third, it is deficient to address the severity based on the Diagnostic and Statistical Manual of Mental Disorders, Fifth Edition. Fourth, this study did not include misuse of other substances that could have negative effects in addition to the use of illegal drugs and tobacco products. Substance abuse patterns and sociodemographic characteristics were detailed, including psychiatric comorbidities and the most commonly used substances. These data might decrease the knowledge gap in Makkah City. We recommend more studies about this knowledge gap and our study limitations. There also should be a focused scope on psychiatric comorbidities.

## Conclusions

Substance use has a harmful impact worldwide on various levels among individuals, families, financial, occupation, and socioeconomic status. According to the articles that have been published, the Kingdom of Saudi Arabia address the same concerns and impact. This study showed the pattern of substance abuse in Makkah City and related psychiatric etiologies, which will help further studies to highlight the influence of each substance and the clinical consequences among Makkah residents. Among the most commonly abused substances in Makkah, 28.2% of the participants used different forms of nicotine, followed by cannabis (2.4%) and khat (2.2%). Meanwhile, the prevalence of other substances, such as sedatives/hypnotics, alcohol, opioids, amphetamines, methamphetamines, ecstasy, and cocaine, is 1.7%, 1.4%, 0.6%, 0.4%, 0.3%, 0.3%, and 0.1%, respectively. Substance abuse positively correlates with sociodemographic data, with males having a higher risk, and psychiatric neurosis is associated with childhood trauma and anxiety.
